# Repeating Patterns of Mimicry

**DOI:** 10.1371/journal.pbio.0040341

**Published:** 2006-10-17

**Authors:** Axel Meyer

## Abstract

Axel Meyer discusses how evolution frequently converges on similar mimetic patterns in different species.

Fascination with flora and fauna usually starts early in life as an all-encompassing childhood pastime. Growing up in Germany in the 1960s and 1970s, I developed an affinity for natural history as a child, inspired by famous television naturalists, such as Jacques Cousteau and Bernhard Grzimek, as well as by role models closer to home. As a child, it seemed quite natural to observe, experiment with, and collect all kinds of animals, dead or alive, and their parts (beetles, butterflies, fish, amphibians, antlers, and skulls) for my private “Wunderkammer,” or cabinet of curiosities.

Literature Nobel laureate Vladimir Nabokov, probably most famous for his notorious novel Lolita, was also a distinguished lepidopterist who specialized in the systematics of the butterfly family Lycaenidae. Nabokov's obsession with butterflies also started early in life and arguably influenced his thinking and writing for the rest of it. He published numerous scholarly papers in recognized entomology journals, mostly on species from Europe and North America, and he was inordinately proud that a species of butterfly (Cyllopsis pyracmon nabokovi) was named after him. Nabokov was particularly fascinated by the mapping of spots on butterfly wings.

The diversity of life, both within and between species, often lights the fire of childhood fascination, fostering a naïve juvenile obsession and the desire to collect. But as one becomes more familiar with the natural world, one also notices curious similarities between organisms. This similarity might simply, and rather uninterestingly, be a reflection of close evolutionary relationships. But more intriguing evolutionary questions and lessons emerge when the similarity exists between distantly related species. Convergence, the term used to describe this type of similarity, is arguably more interesting than plain diversity. The paleontologist Simon Conway Morris [1] has made convergence his main theme, because it not only highlights the power of natural selection but also helps to identify other mechanisms of evolution that will constrain and canalize the possibilities of diversity into a reduced subset of similar evolutionary outcomes. How phenotypic diversity is constrained by genetics or development, and more specifically, what underlying processes determine phenotypic outcomes are exciting questions that the nascent field of evolutionary developmental biology addresses.

Convergence teaches us that the seemingly unbounded creativity of evolution through natural selection and the diversity it produces are not without limits but sometimes seem to follow predictable patterns verging on rules. This is also evident in one of the prime examples of diversity in vertebrates, the species flocks of cichlid fishes of Eastern Africa. Although each of the three large East African Lakes contains hundreds of endemic species of cichlids [2,3], the observed diversity in each lake is often strikingly repeated between the adaptive radiations of these largely reciprocally monophyletic (evolutionarily distinct) groupings that make up these species flocks [4,5]. It is astonishing that one not only finds certain types of ecological guilds—such as algae scrapers, fish eaters, snail crushers, and zooplankton pickers—in each of the lakes, but that sometimes even the color patterns of these phylogenetically rather distantly related species from different lakes match in a striking fashion [5].

Redundancy in the outcome of natural selection is most evident in the beautiful examples of mimicry in butterflies (Figure 1). Mimicry comes in different forms. One is between poisonous and nonpoisonous butterfly species, discovered by and named after the British naturalist Henry Walter Bates. Here, a harmless palatable mimic species derives a selective advantage from its similarity to the poisonous model species; a predator might spare the mimic if it had learned to avoid butterflies with a certain color pattern from an earlier unpleasant noxious encounter with the unpalatable model. In Müllerian mimicry—named after its discoverer, the German zoologist Fritz Müller—several equally unpleasantly tasting species share a color pattern, and all species benefit mutually, not only the mimic.

**Figure 1:**
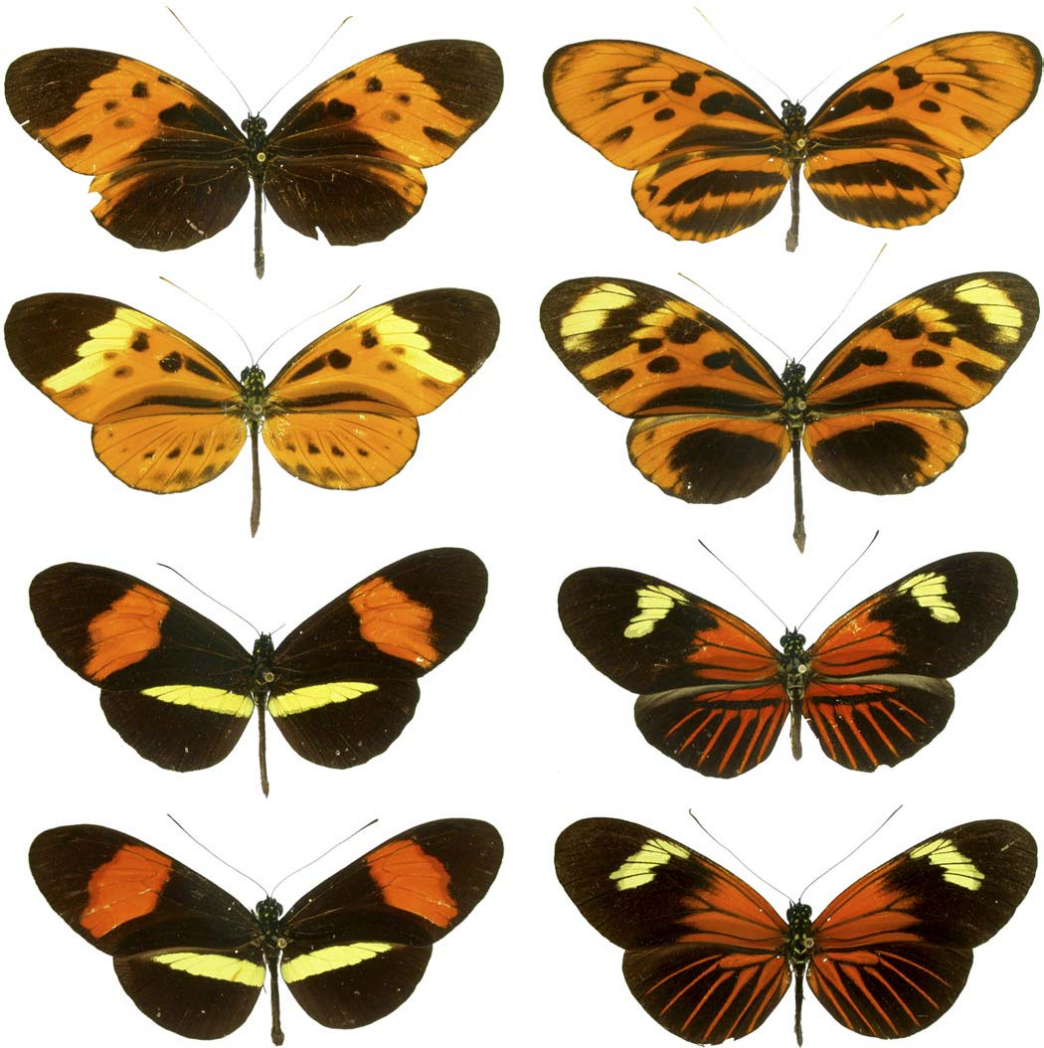
Mimicry in Butterflies Is Seen here on These Classic “Plates” Showing Four Forms of H. numata, Two Forms of H. melpomene, and the Two Corresponding Mimicking Forms of H. erato This highlights the diversity of patterns as well as the mimicry associations, which are found to be largely controlled by a shared genetic locus [15].

Evolution clearly repeats itself in mimicry. The “why” and “how” is at the center of a field of research that combines comparative genomics and evolutionary developmental approaches. Through a variety of technical approaches in comparative developmental genetics and the construction of genetic linkage maps that can identify chromosome regions and sometimes even specific genes that control phenotypic traits, it has now become possible to bridge the wide gap between natural history and developmental biology. During the past decade, the development and evolution of color patterns in butterfly wings has become one of the best-studied examples in which knowledge from molecular developmental biology—with lessons and tools from Drosophila developmental genetics—paved the way and has led to a deeper understanding of both the genetic underpinnings and the evolutionary implications of butterfly color evolution. The Bicyclus system from Africa analyzed by Paul Brakefield from Leiden University (Leiden, Netherlands) and Sean Carroll from the University of Wisconsin Madison (Madison, Wisconsin, United States) [6–9], and the classical Papilio mimicry system investigated by Frederik Nijhout from Duke University [10] (Durham, North Carolina, United States) both continue to provide beautiful examples for the ways in which developmental underpinnings and molecular mechanisms interact with natural selection.

The Heliconius butterflies from the tropics of the Western Hemisphere (Figure 1) are the classical model for Müllerian mimicry, and during the last decade, several laboratories [11] have studied their natural history [12]. Also in recent years, the developmental and genetic basis of the convergent mimetic wing patterns of several co-model Müllerian mimicry systems have been investigated [13]. Through the comparative genetic linkage analyses of several of these crosses, it was even possible to link the genomic region for the matching color patterns (the “mimicry locus”) with that for mate preference [14], hinting at the possibility that these color genes may be “speciation genes” that are directly involved in the evolution of reproductive isolation.

Two species of butterflies, H. erato and H. melpomene, have each evolved into more than 20 different geographic races. These races have converged to display similar markings between the two species due to Müllerian mimicry. Because they can be crossed, the races offer an excellent opportunity to study the genetic basis of phenotypic adaptation, diversification, and mimetic convergence. As reported by Joron et al. [15] in this issue of *PLoS Biology,* the across-species investigation of the developmental basis from three species of Heliconius wing diversity, or in this case, similarity (or even more precisely, convergence) yielded very interesting new results. The crosses, genomic analyses of bacterial artificial chromosome clones, and genetic maps demonstrate that color pattern divergence has a relatively simple genetic basis—few regions of the genome control most of the dramatic color-pattern differences between races. That the same genetic region is used across species boundaries to create similar (mimetic) colors in some and divergent patterns in other species demonstrates that phenotypic similarity (and divergence) can be potentially controlled by the same set of genes.

Joron et al. [15] present data from a genetic linkage map of the genome of Heliconius butterflies that show that a locus, *Yb*, controls the presence of a yellow wing band in H. melpomene. Interestingly, the *Yb* locus maps to the same genomic location as the Cr locus in its congener and co-mimic H. erato, which also sports a yellow band on its wings. Evolution appears to have recruited the same gene region repeatedly and in parallel, resulting in the similar phenotypes of these Müllerian mimics. Natural selection apparently pushed for similar phenotypes, yet the conservative nature of the underlying developmental genetic mechanisms constrained (in the sense that the same genes and pathways are used) the outcome of selection as well.

What makes this study [15] even more fascinating is that in a third species with variable coloration, H. numata—in which some morphs can look quite different from both H. melpomene and H. erato—the same genetic locus appears to be involved in bringing about a phenotype that mimics that of the distantly related butterflies of the genus Melinaea. So, although this gene locus is involved in similar wing color patterns in H. melpomene and its co-mimic H. erato, it acts as a “supergene” in conjunction with other unlinked color-pattern loci to control the development of quite different wing color patterns in different populations of H. numata. It is remarkable and warrants further investigation that the same genomic region is controlling not only convergent wing color patterns in H. erato and H. melpomene, but also divergent patterns in H. numata. So, genomic conservatism at an upstream supergene and seemingly unconstrained downstream genes combined to simultaneously produce mimetic similarity and novel color patterns across species of butterflies.

Future comparative genomic work, for example on expressed sequence tags and DNA microarrays of the developing wings of different Heliconius species, could be used to identify the expected downstream target genes of this supergene. These approaches might reveal how the genetic architecture of downstream genetic pathways, which are involved in the expression of similar or divergent color patterns during wing development, might have diverged and whether some of their components show signatures of Darwinian selection. As Joron and co-workers' investigation [15] beautifully illustrates, the study of the genetics of adaptive features is perhaps the field of evolutionary inquiry that will yield the most interesting new insights into large-scale evolutionary diversification.

## References

[pbio-0040341-b001] Conway Morris S (2003). Life's solution.

[pbio-0040341-b002] Meyer A, Kocher TDK, Bassasibwaki P, Wilson AC (1990). Monophyletic origin of Lake Victoria cichlid fishes suggested by mitochondrial DNA sequences. Nature.

[pbio-0040341-b003] Verheyen E, Salzburger W, Snocks J, Meyer A (2003). The origin of the superflock of cichlid fishes from Lake Victoria, East Africa. Science.

[pbio-0040341-b004] Meyer A (1993). Phylogenetic relationships and evolutionary processes in East African cichlids. Trends Ecol Evol.

[pbio-0040341-b005] Stiassny MLJ, Meyer A (February 1999). Cichlids of the African rift lakes. Scientific American.

[pbio-0040341-b006] Brakefield PM, Gates J, Keyes D, Kesbeke F, Wijngaarden P (1996). Development, plasticity, and evolution of butterfly eyespot patterns. Nature.

[pbio-0040341-b007] Brunetti CR, Slegue JE, Monteiro A, French V, Brakefield PM (2001). The generation and diversification of butterfly eyespot color patterns. Curr Biol.

[pbio-0040341-b008] Brakefield PM (2006). Evo-devo and constraints on selection. Trends Ecol Evol.

[pbio-0040341-b009] Gompel N, Prud'homme B, Wittkopp PJ, Kassner VA, Carroll SB (2005). Chance caught on the wing: cis-regulatory evolution and the origin of pigment patterns in Drosophila. Nature.

[pbio-0040341-b010] Nijhout HF (1991). The development and evolution of butterfly wing patterns.

[pbio-0040341-b011] Brower AVZ (1996). Parallel race formation and the evolution of mimicry in Heliconius butterflies: A phylogenetic hypothesis from mitochondrial DNA sequences. Evolution.

[pbio-0040341-b012] Kapan DD (2001). Three-butterfly system provides a field test of Müllerian mimicry. Nature.

[pbio-0040341-b013] Kapan DD, Flanagan NS, Tobler A, Papa R, Reed RD (2006). Localization of Müllerian mimicry genes on a dense linkage map of Helconius erato. Genetics.

[pbio-0040341-b014] Kronforst MR, Young LG, Kapan DD, McNeely C, O'Neill RJ (2006). Linkage of butterfly mate preference and wing color preference cue at the genomic location of wingless. Proc Natl Acad Sci U S A.

[pbio-0040341-b015] Joron M, Papa R, Beltrán M, Chamberlain N, Mavárez J (2006). A conserved supergene locus controls colour pattern diversity in Heliconius butterflies. PLoS Biol.

